# Inhibitory Phosphorylation of Separase Is Essential for Genome Stability and Viability of Murine Embryonic Germ Cells

**DOI:** 10.1371/journal.pbio.0060015

**Published:** 2008-01-29

**Authors:** Xingxu Huang, Claudia V Andreu-Vieyra, J. Philippe York, Rashieda Hatcher, Tao Lu, Martin M Matzuk, Pumin Zhang

**Affiliations:** 1 Department of Molecular Physiology and Biophysics, Baylor College of Medicine, Houston, Texas, United States of America; 2 Department of Pathology, Baylor College of Medicine, Houston, Texas, United States of America; 3 Department of Experimental Therapeutics, University of Texas MD Anderson Cancer Center, Houston, Texas, United States of America; 4 Department of Biochemistry and Molecular Biology, Baylor College of Medicine, Houston, Texas, United States of America; Harvard Medical School, United States of America

## Abstract

Activity of separase, a cysteine protease that cleaves sister chromatid cohesin at the onset of anaphase, is tightly regulated to ensure faithful chromosome segregation and genome stability. Two mechanisms negatively regulate separase: inhibition by securin and phosphorylation on serine 1121. To gauge the physiological significance of the inhibitory phosphorylation, we created a mouse strain in which Ser1121 was mutated to Ala (S1121A). Here we report that this S1121A point mutation causes infertility in mice. We show that germ cells in the mutants are depleted during development. We further demonstrate that S1121A causes chromosome misalignment during proliferation of the postmigratory primordial germ cells, resulting in mitotic arrest, aneuploidy, and eventual cell death. Our results indicate that inhibitory phosphorylation of separase plays a critical role in the maintenance of sister chromatid cohesion and genome stability in proliferating postmigratory primordial germ cells.

## Author Summary

Higher eukaryotes rely on a separate cell lineage, the germline, to pass genetic information from generation to generation. To ensure faithful transmission of genetic information, cell cycle checkpoint mechanisms are engaged during mitotic and meiotic divisions of germ cells. The identity and function of these checkpoints is not well understood. In mammals, the germline is specified early in embryogenesis as primordial germ cells (PGCs) at the epiblast stage (around embryonic day 5.0 in mice). PGCs then migrate out from their birthplace and arrive at the genital ridge several days later. In the genital ridge, PGCs undergo a great expansion in number through mitosis. During this expansion, PGCs critically depend on the inhibitory phosphorylation of separase to prevent premature separation of sister chromatids and hence progeny with abnormal chromosome number. Separase is a protease which cleaves the Scc1 subunit of sister chromatid cohesin complex. Its activity must be suppressed before all sisters are aligned at the metaphase plate. Two mechanisms are known that can inhibit separase: phosphorylation and binding by securin, both of which are activated at the spindle assembly checkpoint. Although these two mechanisms are redundant in somatic cells, our results indicate that the inhibitory phosphorylation of separase is uniquely required in the germline.

## Abstract 3

A single point mutation of separase that blocks its phosphorylation has a profound and dominant effect on germ cell biology.

## Introduction

Faithful transmission of duplicated genetic material is of fundamental importance to the viability of all organisms. In eukaryotes, sister chromatids are closely connected by cohesin complexes established during S phase. The core cohesin complex is composed of the protein subunits Smc1, Smc3, Scc1, and Scc3 [1] and is believed to form a ring-like structure enclosing the two sister chromatids [2]. Prior to anaphase, the majority of cohesin on chromosomal arms is removed by Plk1- and Aurora B–mediated phosphorylation of cohesin subunit Scc3 [3–7]. However, the final separation of sister chromatids in anaphase depends on separase-mediated cleavage of Scc1 [8,9]. To prevent premature separation of sister chromatids, separase must be tightly regulated. In yeast, this occurs through direct inhibition by securin [10]. In vertebrates, inhibitory phosphorylation of separase provides an additional layer of regulation [11]. In humans, phosphorylation of Ser1126 and Thr1326 inhibits separase activity by allowing Cdk1/cyclin B1 to bind and inhibit the protease [12].

Loss of *securin* is lethal in fission yeast and *Drosophila* [13–15] and causes chromosomal instability in budding yeast [10]. However, *securin*-deficient mice are viable and apparently normal [16]. Mammalian cells lacking *securin* do not show obvious growth defects and maintain sister chromatid cohesion when challenged with spindle microtubule poisons [16–18]. These results suggest redundancy in the inhibition of separase by *securin* and by inhibitory phosphorylation. Indeed, our previous studies demonstrate that murine embryonic stem cells carrying a nonphosphorylatable separase allele and with *securin* deleted are sensitive to nocodazole and cannot maintain sister chromatid cohesion in response to microtubule disruption [19]. However, we do not know whether these two separase-regulating mechanisms are redundant at the level of the organism or if the inhibitory phosphorylation of separase plays any role in development.

Infertility in humans has a strong genetic contribution. It is estimated that genetic etiology is responsible for approximately 15% male and 10% female sterility [20], which certainly is an underestimation because many of the idiopathic sterilities in clinic may have unidentified genetic causes. Chromosome abnormalities or aneuploidy in germ cells are often cited as the cause for failed conceptions. Aneuploidy can result from errors in mitosis or meiosis of germ cells. Yet, we know little about the molecular mechanisms that ensure genome stability in germ cells, during their development, and in meiosis. In this study, we report the analysis of mice carrying a nonphosphorylable *separase* allele. We show that these mice are sterile due to the depletion of germ cells during embryogenesis, demonstrating a unique role of inhibitory phosphorylation of separase in germline development.

## Results

### Generation of Mice Carrying a Nonphosphorylatable *separase* Allele

Mouse *separase* is encoded by 31 exons spanning about 90 kb on Chromosome 15 near the telomere. The two inhibitory phosphorylation sites Ser1121 (Ser1126 in human) and Thr1341 (Thr1346 in human) are located in exon 18. Because phosphorylation of Ser1121 contributes the most to separase inhibition [11], we reasoned that mutating this serine residue to alanine would destroy this separase-inhibiting mechanism. We first generated a number of mouse embryonic stem cell clones in which one *separase* allele was modified by introducing the *S1121A* point mutation through homologous recombination [19]. We selected for the knock-in through a floxed *Puro* marker in intron 17 and named this allele *S1121A-flox-Puro separase*. The point mutation is expressed only after the removal of the *Puro* marker through the action of Cre recombinase. The modified allele is dominant, and when combined in ES cells with a *securin* deletion, causes the failure of sister chromatid cohesion upon treatment with nocodazole [19]. The *S1121A-flox-Puro separase* allele was successfully transmitted through the germ line. The resulting *separase^+/S1121A-flox-Puro^* mice are normal and fertile. Intercrosses of *separase^+/S1121A-flox-Puro^* mice demonstrated that the homozygous *S1121A-flox-Puro separase* allele is lethal, and the embryos died at the same developmental stage as reported for *separase* knockouts [21,22], suggesting that *S1121A-flox-Puro separase* is a null allele. To activate the point mutation, we crossed *separase^+/S1121A-flox-Puro^* mice with *Meox2^+/Cre^* mice which express *Cre* as early as 5.5 days post-conception (dpc) in the epiblast, causing Cre-mediated recombination in most lineages including the germ line [23]. Thus, the resulting *Meox2^+/Cre^Separase^+/S1121A-flox-Puro^* mice are essentially *Meox2^+/Cre^Separase^+/S1121A^* because the vast majority of the cells in these animals should have the *Puro* marker deleted from the *separase* locus by Cre-mediated recombination. PCR genotyping using tail DNA confirmed the conversion from *S1121A-flox-Puro* to *S1121A*.

### *Separase^+/S1121A^* Mice Are Sterile

*Meox2^+/Cre^Separase^+/S1121A^* mice are indistinguishable from their littermates, which are *Meox2^+/+^Separase^+/+^*, *Meox2^+/+^Separase^+/S1121A-flox-Puro^*, or *Meox2^+/Cre^Separase^+/+^*. They do not show any overt abnormalities up to 2 y of age. However, both male and female *Meox2^+/Cre^Separase^+/S1121A^* mice are sterile. To rule out the possibility that sterility in the mutant mice is a combined effect of the *separase* S1121A point mutation and *Meox2* heterozygosity (due to the *Cre* knock-in), we used another *Cre* strain, *EIIa-Cre*, in which *Cre* is expressed as early as the zygote stage [24]. *Separase^+/S1121A^/EIIa-Cre* mice were also infertile (unpublished data), indicating the infertile phenotype in the mutant mice is caused by the *separase* point mutation rather than by heterozygosity for *Meox2* or an effect of *Cre*. Because of sexual dimorphism arising from the *separase* point mutation, the analysis of the female phenotype will be reported elsewhere.

**Figure 1:**
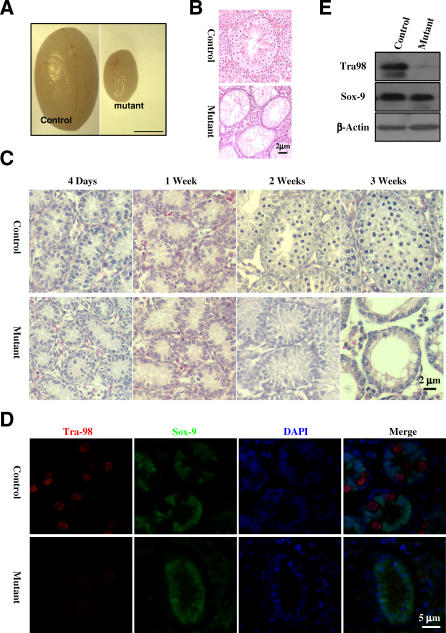
Lack of Spermatogonia in Nonphosphorylatable *separase* S1121A Mutant Mice (A) Testes from 6-wk-old control and mutant littermates. (B) Micrographs of hematoxylin and eosin–stained sections of testes in (A). (C) Micrographs of hematoxylin and eosin–stained sections of testes isolated from animals at different ages. (D) Immunofluorescence staining of Tra98 (red) and Sox-9 (green). Sections of 4-d-old testes were used. Nuclei were counterstained with DAPI (blue). (E) Western blot analysis of Tra98 and Sox-9 using 2-wk-old testes.

**Figure 2:**
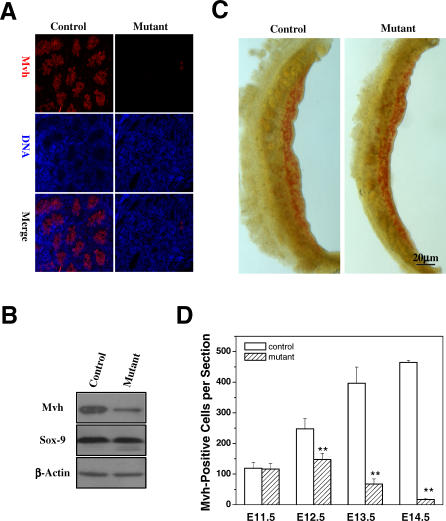
Loss of Fetal Germ Cells in Nonphosphorylatable *separase* S1121A Mutant Mice (A) Immunofluorescence staining of Mvh using 14.5 dpc testes. (B) Western blot analysis of Mvh and Sox-9 expression in 13.0 dpc gonads. (C) Whole-mount AP staining of 11.5 dpc genital ridges. AP-positive cells are red. (D) Quantitation of Mvh-positive cells in the control and mutant gonads. Serial sections of male genital ridge/testes were stained for Mvh. The Mvh-positive cells were scored from at least three sections in at least three embryos (six genital ridges or testes) for each time point. The mean value is shown with the standard errors. (* denotes *p* < 0.05 and ** *p* < 0.001).

**Figure 3:**
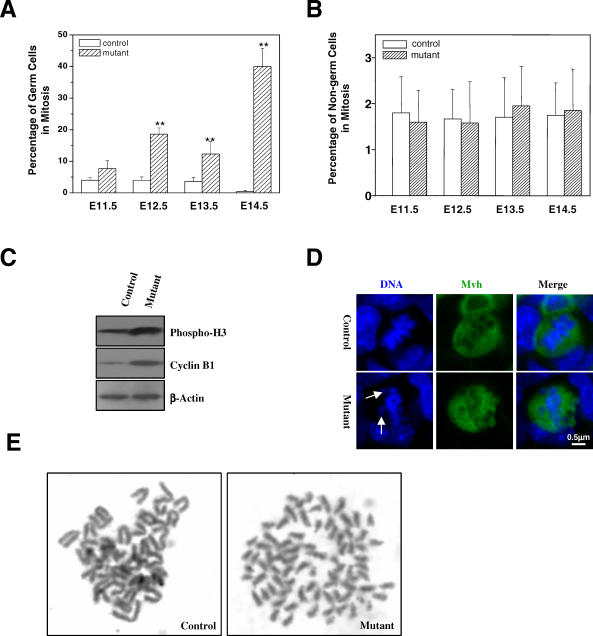
Mitotic Arrest of *separase* S1121A Mutant Germ Cells (A) Mitotic indices of germ cells at different stages of development. (B) Mitotic indices of non–germ cells at different stages of development. (C) Western blot analysis of phosphorylated histone H3 in extracts of 13.5 dpc genital ridges. (D) Abnormal metaphase configuration of mutant germ cells at 13.5 dpc. Arrows indicate misaligned chromosomes. (E) Chromosome spreads of cultured PGCs after 6-h nocodazole treatment.

At 6 wk of age, mutant testes are much smaller than the controls (Figure 1A). Histological examination revealed that the mutant seminiferous tubules are devoid of spermatocytes and spermatids (Figure 1B), suggesting spermatogenesis failure in *separase* S1121A mutant mice. Spermatogenesis in mice starts around 10 d post-partum (dpp) when spermatocytes are first produced, followed by spermatids around 20 dpp and spermatozoa around 35 dpp [25]. We examined testes isolated from mice ranging from 4 dpp up to 2 wk old. We could not detect histologically any differences between the control and the mutant before the age of 2 wk (Figure 1C). However, at 2 wk when the first wave of spermatogenesis had started in the control, we found no sign of spermatogenesis in the mutant (Figure 1C). The most likely reason for this result is the lack of spermatogonia in the mutant testes. To determine if that was the case, we carried out immunostainings for Tra98, a germ cell marker [26], and Sox9, a marker for Sertoli cells [27,28]. As shown in Figure 1D, we could not detect Tra98 in the mutant testes at 4 d after birth, whereas Sox-9 was readily detected. Absence of Tra98 was confirmed by western blot analysis (Figure 1E). Three additional germ cell markers, Plzf [29], Sohlh1 [30], and GCNA [31] were also absent in the mutant testes (Figures 1–3 and S1–S3), indicating that the loss of inhibitory phosphorylation of separase leads to spermatogonia cell depletion.

### Fetal Germ Cells Are Depleted in *separase^+/S1121A^* Mice

Spermatogonia originate from primordial germ cells (PGCs). PGCs emerge as a group of about 45 founder cells at the base of the future allantois in the epiblast at 7.5 dpc [32–36]. PGCs can be recognized by their high levels of expression of tissue-nonspecific alkaline phosphatase [37]. From 8.5 to 10.5 dpc, PGCs migrate towards the genital ridge and increase their numbers through mitotic divisions [38]. In the mouse, most PGCs enter the genital ridge at 11.5 dpc, lose their locomotive ability, and expand from about 3,000 to over 25,000 cells [32,33,38]. This expansion ends between 13.5 and 14.5 dpc. Immunostaining (Figure 2A) and western blot (Figure 2B) analyses of genital ridges from mutant and control mice at 14.5 dpc showed that *separase* point mutant testes lack the early germ cell marker Mvh (mouse vasa homologue) [39], suggesting the absence of PGCs at this stage.

The germ cell deficiency in *separase* point mutant testes could arise from a failure of PGCs to reach the genital ridge or a failure to proliferate after reaching the genital ridge. To distinguish between these two possibilities, we took advantage of the high expression levels of tissue nonspecific alkaline phosphatase (AP) [37] by PGCs to analyze their fate during migration. At 11.5 dpc, AP staining showed similar number of AP-positive cells in genital ridges from control and mutant mice (Figure 2C), indicating that mutant PGCs are not impaired in migration. However, while control PGCs expanded greatly in the genital ridge, mutant PGCs did not (Figure 2D). Although mutant PGCs increased in number between 11.5 and 12.5 dpc, the increase was significantly smaller than that of control PGCs (Figure 2D) and their number diminished rapidly over time, as determined by Mvh immunostaining. These data indicate that the *separase* phosphorylation mutant S1121A causes proliferation failure in the germ cells between 11.5 and 14.5 dpc when the PGCs proliferate rapidly in the genital ridge. Therefore, the loss of germ cells during embryonic development in *separase* point mutants explains the observed absence of germ cells in the postnatal testes.

### *Separase* Point Mutant Germ Cells Undergo Aberrant Mitoses

Because the point mutation in *separase* could generate a constitutively active enzyme, we reasoned that mutant PGCs might prematurely separate their sister chromatids before the onset of anaphase. The precociously separated sister chromatids would not be able to align at the metaphase plate nor will they generate tension at their kinetochores, leading to activation of the spindle assembly checkpoint and mitotic arrest [40,41]. One prediction of this hypothesis is that mutants would have more PGCs in mitosis than controls due to spindle checkpoint-mediated mitotic arrest. Therefore, we determined the mitotic indices of the germ cells in genital ridges by quantitating the number of Mvh-positive cells that contained condensed chromosomes. As shown in Figure 3A, the mitotic indices of mutant PGCs were slightly higher than controls at 11.5 dpc and were significantly higher than controls at 12.5, 13.5, and 14.5 dpc, whereas the mitotic indices of non–germ cells were the same between the control and the mutant (Figure 3B). We also measured the expression levels of phospho-histone H3 and cyclin B1 in 13.5 dpc genital ridges by western blotting. Again, these two mitotic markers were expressed at higher levels in the mutant than in the control (Figure 3C). Furthermore, we observed that the majority of mutant mitotic figures were abnormal and showed misaligned chromosomes (Figure 3D), suggesting premature separation of sister chromatids. To visualize precocious sister separation, we cultured PGCs isolated from 11.5 dpc genital ridges and treated the cells with nocodazole for 6 h to enrich mitotic cells. As shown in Figure 3E, the mutant PGCs showed complete separation of sister chromatids while the control did not, indicating that inhibitory phosphorylation of separase is essential in maintaining sister chromatid cohesion in PGCs. Taken together, these data demonstrate that the point mutation in *separase* causes precocious sister chromatid separation and the accumulation of PGCs in mitosis due to spindle checkpoint activation.

### Adaptation of Spindle Checkpoint in Mutant Germ Cells

**Figure 4:**
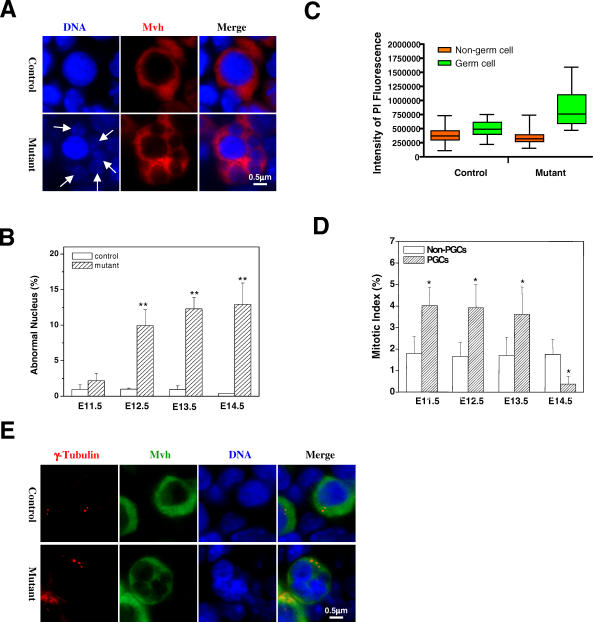
*Separase* S1121A Mutant Germ Cells Undergo Abnormal Mitosis (A) Micronucleus formation in the mutant at 13.5 dpc. Arrows indicate the micronuclei. (B) Quantitation of germ cells with micronuclei at different gestational time points. The mean value from three embryos is shown with standard error (* denotes *p* < 0.05 and ** *p* < 0.001). (C) Aneuploidy in mutant germ cells. Germ cells in sections of genital ridges at 13.5 dpc were stained with anti-Mvh and counterstained for DNA with propidium iodine. The DNA content of both germ and non–germ cells was determined through laser scanning cytometry. (D) Mitotic indices of germ and non–germ cells in controls. The mean value from three embryos is shown with standard error (* denotes *p* < 0.05). (E) Abnormal centrosome number in mutant germ cells at 13.5 dpc. Red dots of γ-tubulin staining indicate centrosomes.

Over time, mammalian cells adapt to or escape from spindle checkpoint and become tetraploid [42]. Cells with compromised spindle checkpoint function often escape from microtubule poison-induced mitotic arrests to enter tetraploid G1 with micronuclei [43,44]. We observed a large number of mutant PGCs with micronuclei (Figure 4A and 4B), most likely formed by the misaligned and prematurely separated chromosomes once the cells adapted to the spindle checkpoint. When the DNA content of the germ and non–germ cells was determined through laser scanning cytometry and averaged without grouping the cells into G1, S, or G2/M phases, we found that control and mutant non–germ cells contained the same amount of DNA with similar statistical distributions (Figure 4C). By contrast, germ cells in controls contained higher levels of DNA compared with somatic cells, suggesting that cell cycle profiles differ between germ cells and somatic cells and that more germ cells than somatic cells are in G2/M phase (Figure 4C). Consistent with this result, the mitotic indices of germ cells were 2-fold higher than those of somatic cells from 11.5 to 13.5 dpc (Figure 4D). In contrast, germ cell DNA content in mutants was much higher compared with controls and was greater than twice the amount in somatic cells (Figure 4C). Given that the difference in cell cycle profiles between somatic cells and germ cells, the average DNA content of somatic cells can be considered as 2N. It follows then that the DNA content in mutant germ cells is greater than 4N, suggesting that some mutant germ cells might have undergone or initiated another round of DNA synthesis as tetraploid cells. Indeed, some of the mutant PGCs with micronuclei contained more than two centrosomes, which were identified through γ-tubulin staining (Figure 4E), strongly supporting the notion that the mutant germ cells had entered another round of cell division. Taken together, these results strongly suggest that *separase* S1121A mutant PGCs undergo premature sister chromatid separation, mitotic arrest, and adaptation to the spindle checkpoint, resulting in aneuploidy. This phenotype resembles what was previously seen in *securin^–/–^separase^+/S1121A^* ES cells released from nocodazole arrest [19] and in HeLa cells overexpressing human *S1126A* mutant *separase* [45].

### Persistent Aurora B Expression in *separase* Point Mutant Germ Cells

**Figure 5:**
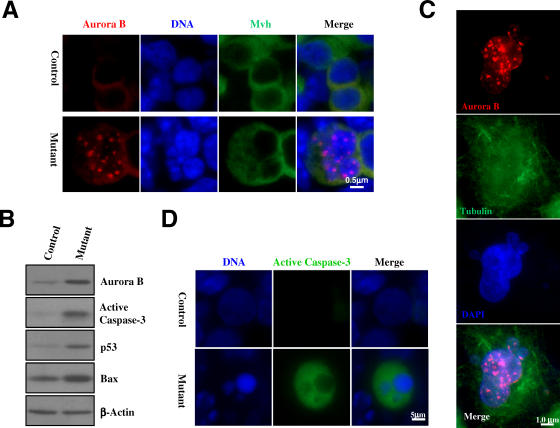
Persistent Expression of Aurora B Kinase in Mutant Germ Cells (A) Immunostaining using antibodies to Aurora B (red) and Mvh (green). Sections of 13.5 dpc gonads were used for the staining. (B) Western blot analysis of Aurora B, active caspase-3, p53, and Bax in extracts of 13.5 dpc genital ridges. (C) Spindle checkpoint–adapted HeLa cells maintain Aurora B expression. HeLa cells were stained for Aurora B (red) and γ-tubulin (green) after 36 h of nocodazole treatment. (D) Immunofluorescence detection of active caspase-3 in mutant genital ridge at 13.5 dpc.

To further demonstrate the mitotic arrest phenotype, we analyzed the expression of Aurora B kinase in PGCs. Aurora B is a chromosome passenger protein required for spindle assembly checkpoint [46,47]. In normal mitosis, Aurora B first associates with the metaphase chromosomes, departs the chromosomes and translocates to the cleavage furrow at anaphase, becomes concentrated in the mid-body during cytokinesis, and finally disappears in G1. When we immunostained with antibodies against Aurora B, we found that this mitotic kinase formed foci in all mutant PGCs with abnormal nuclear morphologies, although the cells were no longer in mitosis as their chromosomes had already decondensed (Figure 5A). Western blot analysis confirmed the immunostaining result (Figure 5B). These data suggest that mutant PGCs adapt to the spindle assembly checkpoint but retain Aurora B expression. To determine if the retention of Aurora B expression is always associated with cells having undergone adaptation to spindle checkpoint, we treated a panel of cell lines with nocodazole, let the cells adapt, and analyzed the pattern of Aurora B expression. All cell lines tested, including HeLa, U2OS, and mouse embryonic fibroblasts, showed the same nuclear Aurora B staining pattern and abnormal nuclear morphologies as did mutant PGCs (Figure 5C, results from HeLa cells are shown). We conclude that the formation of nuclear Aurora B foci is a common feature of mammalian cells adapted to the spindle assembly checkpoint. However, at present, it is unclear if the retention of Aurora B expression is necessary for adaptation.

### Mutant Germ Cells Die by Apoptosis

We next evaluated whether apoptotic cell death could account for the loss of PGCs during embryonic development in *separase* point mutant mice, since apoptosis has been shown to be the type of cell death involved in naturally occurring PGC demise [48]. We found that mutant cells displaying nuclear morphologies consistent with checkpoint adaptation (Figure 4A) were also positive for the apoptotic marker active capase-3 (Figure 5D). Western blot analysis confirmed the presence of higher levels of activated caspase-3 in the mutant genital ridge than in the control (Figure 5B). In addition, we found higher levels of p53 and its pro-apoptotic target Bax in the mutant genital ridge (Figure 5B), suggesting that apoptosis of mutant PGCs is p53-related. In this context, it was reported previously that overexpression of Aurora B can lead to p53 activation [49]. It is therefore plausible that the persistent expression of Aurora B in the mutant PGCs undergoing abnormal mitosis may activate p53, resulting in the elimination of these abnormal germ cells.

### *Securin* Is Not Required for Germline Development

**Figure 6:**
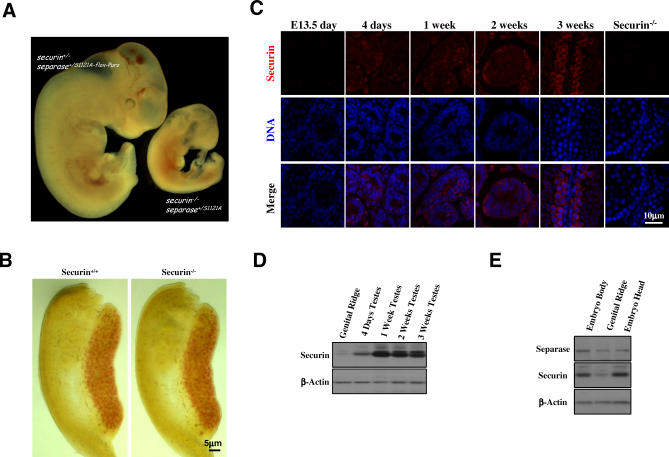
*Securin* Is Not Required for Germline Development (A) Early embryonic lethality in mice lacking both *securin* and the inhibitory phosphorylation of separase. Embryos shown were at 10.5 dpc. The double mutant was much smaller than the control and was retarded in development. (B) No depletion of PGCs is caused by the lack of *securin*. Genital ridges from 12.5 dpc embryos were whole-mount stained for AP activity. (C) Expression of *securin* in different stages of the male germ cell development. Sections of both fetal and postnatal testes were immunostained for securin. Testis from a 3-wk-old *securin^–/–^* mouse was included as a negative control. (D) Western blotting analysis of securin levels in fetal (13.5 dpc) and postnatal testes. (E) Western blotting analysis of separase and securin levels in 13.5 dpc embryos.

The above analyses demonstrate that the inhibitory phosphorylation of separase plays a critical role in the maintenance of male embryonic germ cell genome stability by preventing premature separation of sister chromatids. Besides sterility, mice carrying one *S1121A separase* allele are essentially normal, suggesting redundancy between inhibitory phosphorylation and securin in the regulation of separase in somatic cells. Indeed, elimination of both inhibitory mechanisms causes early lethality (Figure 6A). However, inhibitory phosphorylation is uniquely required for germline development, since lack of *securin* causes no appreciable depletion of germ cells (Figure 6B) nor infertility [16,19]. The question that remains open is the origin of the germline vulnerability to the loss of the inhibitory phosphorylation of separase. One possibility is that post-migratory germ cells express relatively low levels of *securin* so that inhibitory phosphorylation is the primary mechanism that inhibits separase in these cells. To test this possibility, we examined the expression of *securin* in genital ridges. Immunostaining analysis showed that securin levels were undetected in both germ cells and somatic cells (Figure 6C). However, securin was readily detected in postnatal testes (Figure 6C). In agreement with these results, western blotting showed much lower levels of securin expression in the fetal testes than in the postnatal testes (Figure 6D). Furthermore, securin levels in genital ridges were lower than those observed in whole embryos or embryo heads, whereas the level of *separase* expression was similar in fetal genital ridges and other parts of the body (Figure 6E). Since somatic cells in *separase* point mutant genital ridges appear normal and do not display any mitotic defects, these data suggest that the stoichiometric ratio of securin to separase in postmigratory germ cells is lower compared with somatic cells, and these cells must rely completely on the inhibitory phosphorylation of separase to prevent premature activation of the protease.

### Securin and Separase Phosphorylation Are Redundant in PGCs Prior to Their Arrival at the Genital Ridge

**Figure 7:**
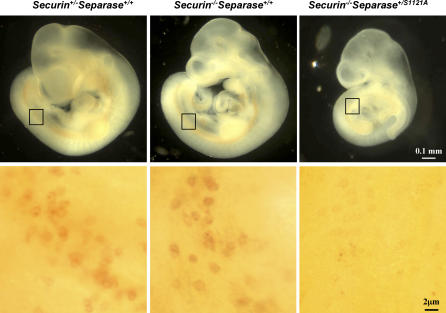
*Securin* and Inhibitory Phosphorylation of separase Are Redundantly Required in Early Germ Cells Embryos at 10.0 dpc were whole-mount stained for AP (top panel). The boxed areas on the embryos were imaged at a higher magnification and shown in lower panel.

The dependence of postmigratory germ cells on inhibitory phosphorylation of separase raises the question of why this mechanism of separase regulation is not required in migratory PGCs. Most likely, it is because of *securin*. To test that, we isolated embryos at 10.5 dpc from crosses between mice that were *securin^+/−^separase^+/S1121A-flox-Puro^* and those that were *securin^−/−^Meox2^+/Cre^* and performed whole mount AP assays on the embryos. As shown in Figure 7, the number of migratory PGCs was the same between *securin^+/−^separase^+/+^* and *securin^−/−^separase^+/+^* mice, indicating that *securin* is not required in these cells, consistent with what we found at a later time (Figure 6B). Strikingly, we could hardly detect any germ cells in the double mutants (Figure 7), demonstrating that securin and the inhibitory phosphorylation are redundantly required in PGCs prior to their arrival at the genital ridge. We do not currently know, however, if the PGCs in the double mutants died during migration, or died shortly after their birth. The exact time of their death needs further investigation.

## Discussion

To prevent unscheduled or precocious sister chromatid separation, separase must be kept inactive prior to anaphase. This is achieved primarily through two negative regulatory mechanisms: securin binding and phosphorylation. In budding yeast, PDS1 (budding yeast securin) is essential for the prevention of sister separation in response to spindle checkpoint [10]. However, we have found that *securin* knockout mice are normal and cells lacking it maintain an intact spindle assembly checkpoint [16]. It was also demonstrated in human cells that *securin* is not required for the maintenance of sister cohesion when microtubule spindle is disrupted [17]. These results indicate that the spindle assembly checkpoint in higher eukaryotes does not solely rely on the inhibition of separase by securin to block sister separation. There must be another mechanism that can keep separase in check, which was found to be phosphorylation [11]. Mammalian separases are phosphorylated at multiple sites. These sites resemble Cdk/MAP kinase phosphorylation sites and are most likely phosphorylated by Cdk1/cyclin B1 in mitosis. More recent work indicates that the phosphorylation itself is not inhibitory, but the phosphorylation is necessary for the binding and inhibition by Cdk1/cyclin B1 complex [12].

Given the redundancy between securin and the inhibitory phosphorylation, it is not surprising that mice carrying a nonphosphorylatable separase allele are essentially normal. However, it is surprising that these mice are sterile. Our analyses demonstrate that the sterility stems from the fact that the postmigratory germ cells are depleted during development in *separase* point mutant mice. What makes the germline particularly vulnerable to this phosphorylation point mutation in *separase*? One possibility is that *securin* may be expressed at relatively low levels in postmigratory germ cells so that stoichiometrically there is more separase than securin. Our results (Figure 6) do support this possibility.

The fact that the number of *separase* S1121A mutant PGCs at 11.5 dpc is about the same as in the control indicates that migratory PGCs are not affected by the mutation. A likely reason is that in the migratory PGCs, securin is still functioning to inhibit separase. Indeed, we found that germ cells were largely eliminated in *securin* and *separase* double mutants (Figure 7), demonstrating a redundancy between *securin* and the inhibitory phosphorylation in migratory PGCs. The redundancy no longer exists in postmigratory germ cells. At present, we do not know why this is the case. Perhaps *securin* levels are higher in migrating PGCs and are reduced once the germ cells take residency in genital ridges. *Securin* overexpression was found highly correlated with the metastatic potential of breast tumors [50], suggesting that the protein might have a role in cell migration separated from its role in inhibiting separase. Since *securin* null PGCs do not display any significant migratory defects, the role of securin (if any) in PGC migration must be nonessential, however. Once arrived at the genital ridge, PGCs may shut off the expression of *securin* along with the entire migratory program, making the postmigratory germ cells vulnerable to nonphosphorylatable separase. Thus, the dependence on the inhibitory phosphorylation of separase is another property that differentiates postmigratory PGCs from migratory PGCs, supporting the notion that PGCs undergo a major phenotypic change once they take residence in the gonads [51], including, perhaps, a switch to low levels of *securin* expression.

Cell cycle control in migratory and postmigratory PGCs is poorly understood. To date, few genes have been shown to negatively affect PGC proliferation in vivo [25]. Our results suggest that postmigratory male PGCs have a strong and functional spindle checkpoint and that the mechanisms governing sister chromatid separation during anaphase in PGC mitoses are distinct from those of somatic cells, and more importantly, from those of migratory PGCs. Although the early germ cell depletion observed in *separase* mutant males precluded studies in meiosis, loss of *separase* in oocytes has been shown to cause failure to resolve chiasma in MI, indicating a requirement for separase in the separation of homolog chromosomes [52]. The relative contribution of securin and the inhibitory phosphorylation to the control of separase activity during meiosis awaits further investigation.

## Materials and Methods

### Mice.

The *S1121A-flox-Puro separase* allele [19] was transmitted through the germline. The resulting *separase^+/S1121A-flox-Puro^* mice were crossed with *Moex2^+/Cre^* mice [23] to generate *Separase^+/S1121A^* mice. Offspring genotyping was performed with PCR analysis using the following primers: for *separase*, pz228a: 5′-cct tct cta acc cag gta gg-3′, pz228b: 5′-aag agc tct acc tac ctc agg-3′, and pz228c: 5′-atc gca tcg agc gag cac gta ctc-3′; for *Meox2*, Cre1: 5′-aag atg tgg aga gtt cgg ggt ag-3′, Cre2: 5′-ggg acc acc ttc ttt tgg ctt c-3′, and Cre3: 5′-cca gat cct cct cag aaa tca gc-3′. pz228a/b amplifies the *S1121A* allele, and pz228b/c *S1121A-flox-Puro*. Embryo sex determination was performed by a PCR analysis of *Sry*, a gene only present on the Y chromosome [53] on genomic DNA.

### Histology and immunofluorescence staining.

Standard histological procedures were followed to prepare testes and fetal gonads for examination. In brief, tissues were fixed in Bouin's solution or 10% neutral buffered formalin (Sigma). The specimens were dehydrated through a graded series of ethanol washes, cleared in Histo-Clear, embedded in paraffin, and sectioned (4 μm thick). The sections were dewaxed, rehydrated, and stained with hematoxylin and eosin. Immunostainings were carried out as described [54].

### Antibodies.

The primary antibodies used were rat anti-GCNA1 (a kind gift of Dr. George C. Enders, University of Kansas Medical Center, Kansas City), rabbit anti-Sox-9 (CHEMICON), mouse anti-β-tubulin (clone E7, Developmental Studies Hybridoma Bank), mouse anti-cyclin B1/mouse anti-p53/rabbit anti-Plzf/rabbit anti-BAX (Santa Cruz Biotechnology), rabbit anti-active Caspase-3/mouse anti-Aurora B (BD Biosciences PharMingen), rabbit anti-phospho-histone H3 (S10) (Cell Signaling Technology), mouse anti-γ–tubulin (Sigma), mouse anti-securin (Novocastra), rabbit anti-Mvh (a kind gift from Dr. Toshiaki Noce, Mitsubishi-Kasei Institute of Life Science, Tokyo, Japan), rabbit anti-Tra-98 (a kind gift from Dr. Yoshitake Nishimune, Osaka University, Japan), and anti-Sohlh1 (from Dr. Aleksandar Rajkovic, Baylor College of Medicine, Houston).

The following secondary antibodies were used: Cy3- or FITC-conjugated anti-rat IgG, Cy3- or FITC-conjugated anti-rabbit IgG, and Cy3- or FITC-conjugated anti-mouse IgG (all from Jackson ImmunoResearch Laboratories).

### Western blot analysis.

Equal amounts of proteins in extracts prepared from testes, gonads, or other tissues were separated with SDS-PAGE and blotted onto polyvinylidene difluoride membrane (Bio-Rad). The blots were probed with the indicated primary and appropriate secondary antibodies (Bio-Rad) and detected with an ECL chemiluminescence kit (GE Healthcare).

### AP staining.

Dissected genital ridges were fixed in 4% paraformaldehyde, washed with PBS, and stained for AP activity with α-naphthyl phosphate (Sigma)/fast red TR (Sigma) solution as described [35].

### Culture of germ cells and chromosome spreads.

11.5 dpc genital ridges were minced and treated with 0.25% Trypsin/EDTA for 30 min at 37 °C to dissociate the cells. The resulting cell suspensions were plated on a feeder layer formed by irradiated LIF-expressing SNL fibroblasts in 15% FBS/DMEM for 2 d. The cells were harvested via trypsin digestion after 6-h nocodazole (65 ng/ml) treatment and subjected to AP staining. AP-positive cells collected through a mouth-pipette were treated for chromosome spread as described [19].

## Supporting Information

Figure S1 Immunofluorescence Staining of Plzf Sections of 1-wk-old testes were used. Nuclei were counterstained with DAPI (blue).
(1.1 MB PPT)

Figure S2 Immunofluorescence Staining of Sohlh1 Sections of 4**-**d-old testes were used. Nuclei were counterstained with DAPI (blue).
(876 KB PPT)

Figure S3 Immunofluorescence Staining of GCNA Sections of 1-wk-old testes were used. Nuclei were counterstained with DAPI (blue).
(724 KB PPT)

## Abbreviations

AP: alkaline phosphatase
dpc: day post-conception
dpp: day post-partum
Mvh: mouse vasa homolog
PGC: primordial germ cell
