# Supplementary figures and images for: Inhibitory Phosphorylation of Separase Is Essential for Genome Stability and Viability of Murine Embryonic Germ Cells
Source: PLoS Biol. 2008 Jan 29;6(1):e15. doi: 10.1371/journal.pbio.0060015 (PMC2214812; doi:10.1371/journal.pbio.0060015)

## Slide 1
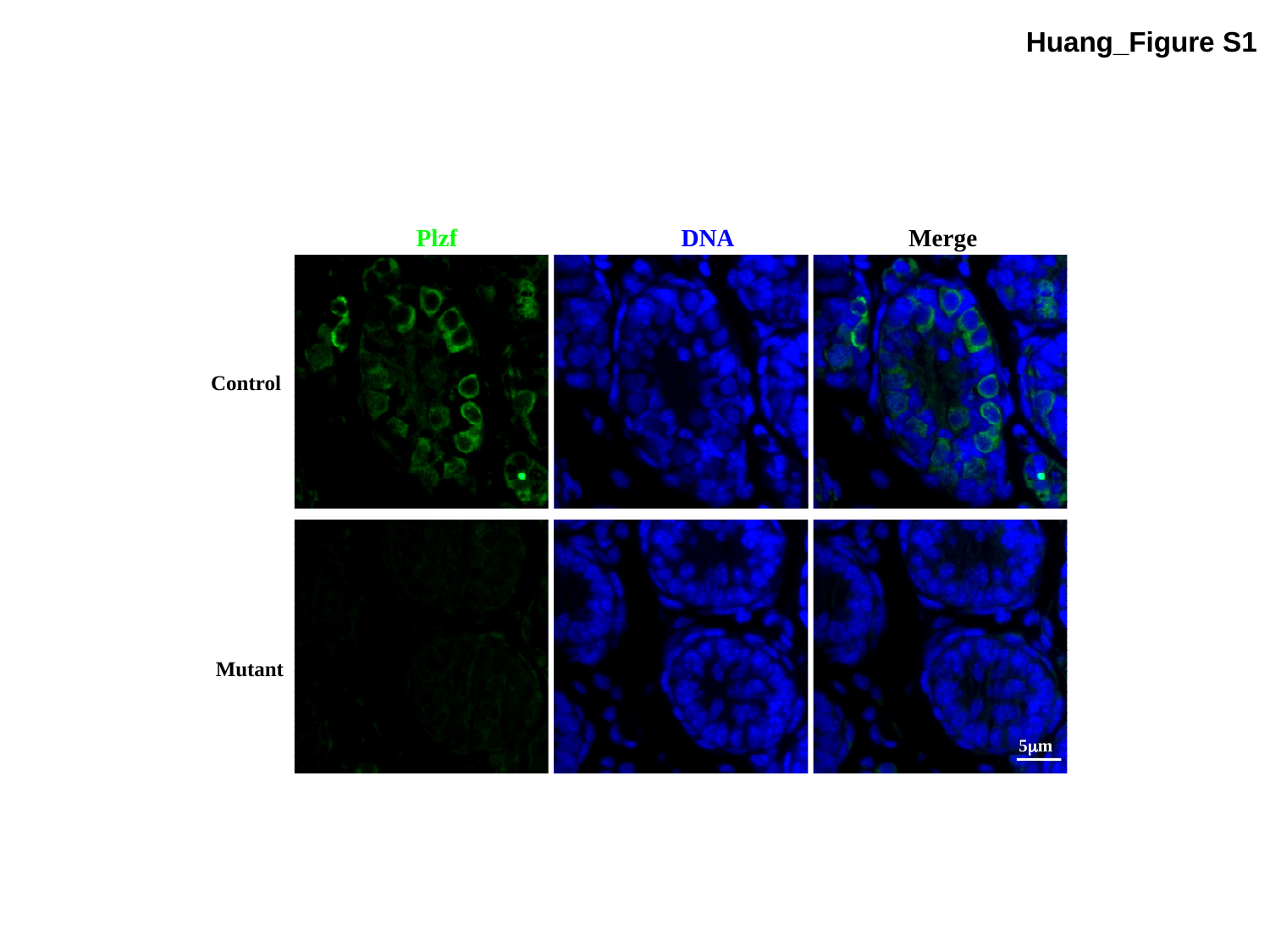

Huang_Figure S1
 Plzf
 DNA
Merge
Control
Mutant
5μm

Supplement: Figure S1 — Sections of 1-wk-old testes were used. Nuclei were counterstained with DAPI (blue). (1.1 MB PPT) [file pbio.0060015.sg001.ppt]

## Slide 1
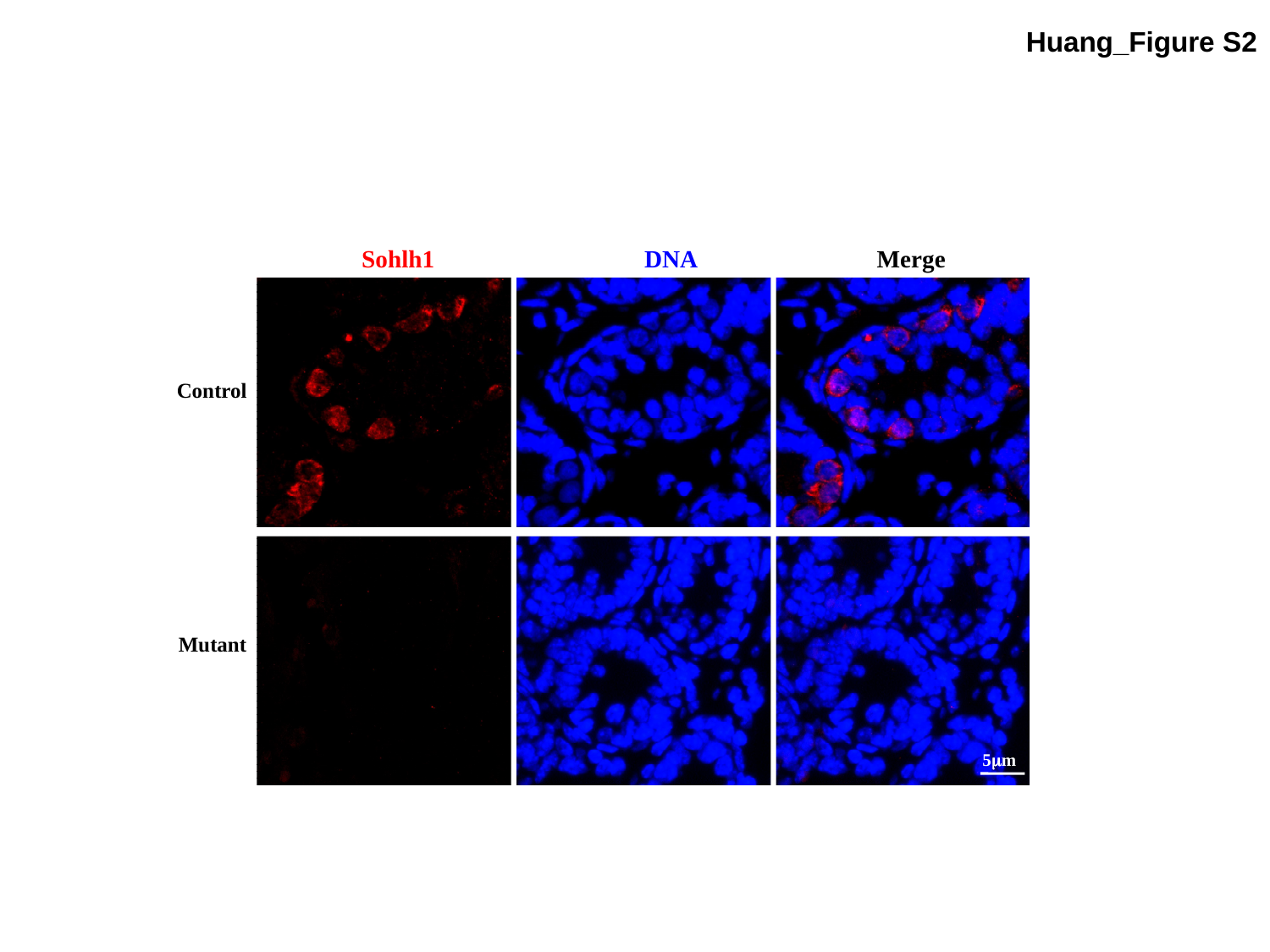

Huang_Figure S2
 Sohlh1
 DNA
Merge
Control
Mutant
5μm

Supplement: Figure S2 — Sections of 4-d-old testes were used. Nuclei were counterstained with DAPI (blue). (876 KB PPT) [file pbio.0060015.sg002.ppt]

## Slide 1
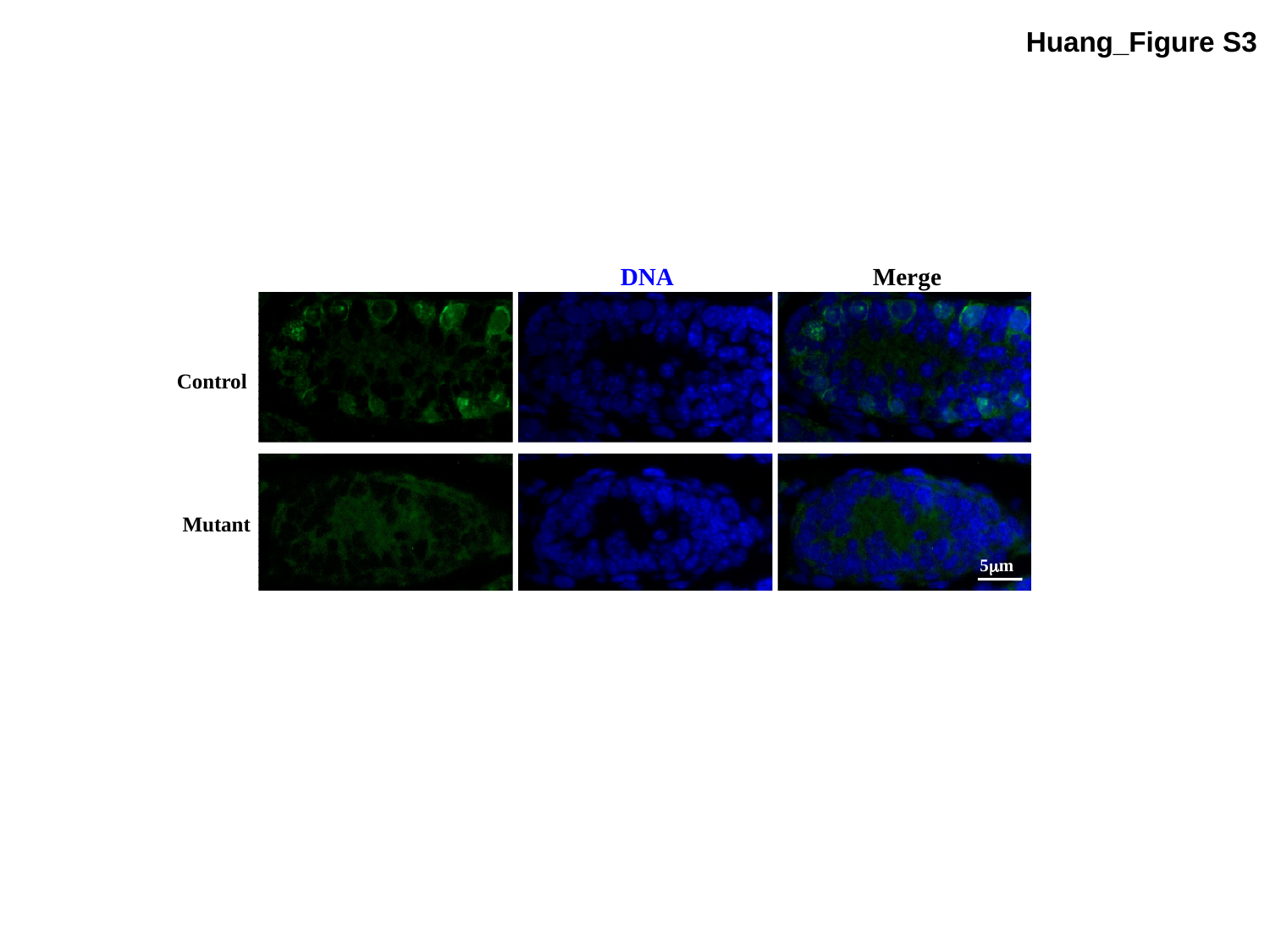

Huang_Figure S3
 GC_x0008_NA
 DNA
 Merge
Control
Mutant
5μm

Supplement: Figure S3 — Sections of 1-wk-old testes were used. Nuclei were counterstained with DAPI (blue). (724 KB PPT) [file pbio.0060015.sg003.ppt]
